# Adaptive and degenerative mitochondrial remodeling define distinct redox states in age-related macular degeneration

**DOI:** 10.1016/j.redox.2026.104365

**Published:** 2026-08-22

**Authors:** Cecilia Giulivi, Carol Villafuerte-Trisolini, Zoe Lee Greenblatt, Jennifer Dang, Atena Tork, Rida Mullah, Sophie Le, Angela Min, Maya Nair, Aldo Vorkapich, Huy-An Tran, Elizabeth Doyle, Alestor Law, Owen O'Keefe, Annabelle Wu, Bradley Shibata, Ana Ripolles-Garcia, Sara M. Thomasy, R. Alan Harris, Rui Chen, Yin Allison Liu, Glenn Yiu

**Affiliations:** a Department of Molecular Biosciences, School of Veterinary Medicine, University of California Davis, Davis, CA, USA; b MIND Institute, University of California at Davis Medical Center, Sacramento, CA, USA; c Department of Ophthalmology, University of California Davis, Sacramento, CA, USA; d Biological Electron Microscopy Facility, University of California, Davis, CA, USA; e Department of Surgical & Radiological Sciences, School of Veterinary Medicine, University of California, Davis, CA, USA; f Human Genome Sequencing Center and Department of Molecular and Human Genetics, Baylor College of Medicine, Houston, TX, USA; g Department of Ophthalmology, Center for Translational Vision Research, University of California, Irvine School of Medicine, Irvine, CA, USA

**Keywords:** Mitochondria, Age-related macular degeneration, Rhesus macaques, Nonhuman primates, Redox homeostasis, Retinal pigment epithelium

## Abstract

Age-related macular degeneration (AMD) is associated with mitochondrial dysfunction and oxidative stress, yet the relationship between mitochondrial remodeling, redox homeostasis, and disease progression remains poorly understood. Nonhuman primates (NHPs) develop spontaneous AMD-related phenotypes, including punctate deposits and soft drusen, providing a unique animal model to investigate mitochondrial pathology in the aging retinal pigment epithelium (RPE).

We integrated quantitative mitochondrial ultrastructural profiling with flavoprotein fluorescence imaging, plasma metabolomics, and whole-exome sequencing to characterize mitochondrial and redox alterations in aged rhesus macaques with AMD-related lesions. Flavoprotein fluorescence imaging demonstrated increased metabolic heterogeneity in eyes with soft drusen, consistent with altered mitochondrial redox states and oxidative stress. Morphometric analysis identified distinct mitochondrial remodeling patterns across phenotypes. Normal aging was characterized by concentric cristae and type I paracrystalline inclusions. Eyes with punctate deposits exhibited increased mitochondrial fusion-associated morphology, hyperbranching, and type I paracrystalline inclusions, consistent with a stress-responsive mitochondrial remodeling pattern. In contrast, eyes with soft drusen exhibited reduced fusion-associated morphology, reduced structural complexity, and ultrastructural features consistent with mitochondrial deterioration. These ultrastructural patterns were accompanied by distinct plasma metabolomic signatures. Punctate deposits were associated with altered glycolytic, tricarboxylic acid cycle, and redox-buffering metabolites, consistent with differences in stress-responsive metabolism, whereas soft drusen exhibited metabolomic signatures consistent with altered redox homeostasis. Whole-exome sequencing identified a mitochondrial DNA variant, MT:9582G > A, in cytochrome *c* oxidase subunit III (*COX3*) associated with the drusen phenotype.

Collectively, these findings identify distinct mitochondrial remodeling patterns associated with AMD-related phenotypes in aged rhesus macaques. The convergence of ultrastructural, imaging, metabolomic, and genetic analyses suggests that punctate deposits and soft drusen are associated with different mitochondrial and redox-related responses to chronic retinal stress. These findings provide a framework for future studies investigating mitochondrial biology and redox-driven mechanisms in AMD.

## Highlights

• Distinct mitochondrial-redox states characterize AMD lesions in macaques.
• Punctate deposits exhibit adaptive mitochondrial remodeling.
• Soft drusen are associated with mitochondrial degeneration.
• Metabolomics reveals divergent redox signatures across phenotypes.
• A *COX3* variant is associated with the drusen phenotype.

## Graphical abstract

**Figure:**
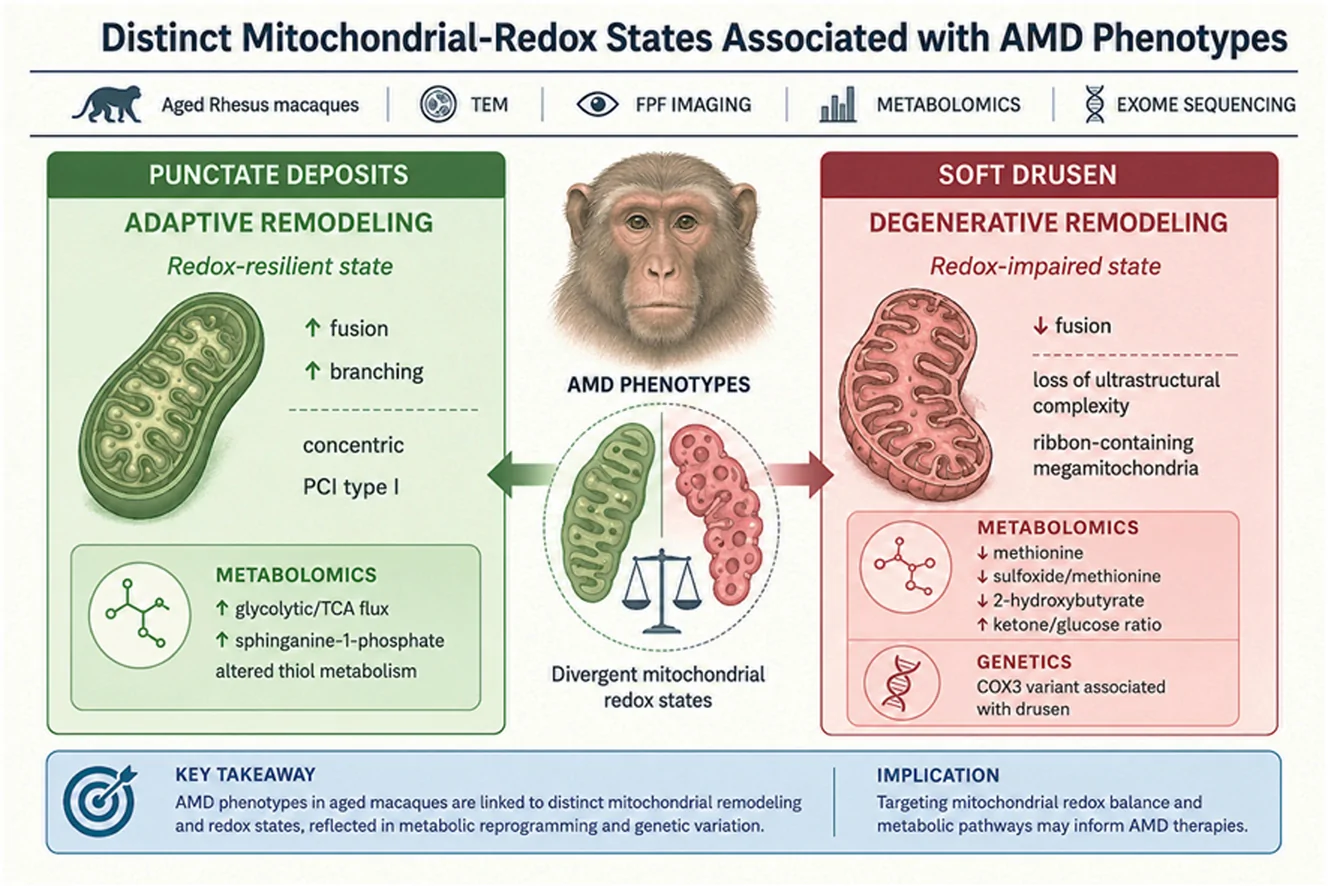

## 1 Introduction

Age-related macular degeneration (AMD) is the leading cause of irreversible vision loss among older adults and is characterized by progressive degeneration of the central retina [1]. Early disease is marked by the accumulation of lipid-rich deposits beneath the retinal pigment epithelium (RPE), known as drusen, which can ultimately progress to geographic atrophy (GA) or macular neovascularization (MNV) [2]. Although AMD is a multifactorial disorder influenced by genetic and environmental risk factors, increasing evidence implicates mitochondrial dysfunction and oxidative stress as central drivers of disease pathogenesis. Chronic redox imbalance at the interface between photoreceptors, RPE, and choriocapillaris promotes lipid peroxidation, inflammation, and cellular dysfunction, suggesting that impaired mitochondrial homeostasis may represent a critical convergence point for multiple AMD-associated pathways.

The RPE is among the most metabolically active tissues in the body and relies heavily on mitochondrial oxidative phosphorylation to support photoreceptor function, visual cycle activity, and outer segment phagocytosis [3]. To sustain these functions, RPE mitochondria must continuously adapt to high oxygen consumption, intense metabolic demand, and lifelong oxidative stress. Under physiological conditions, mitochondrial reactive oxygen species (ROS) serve as signaling molecules that coordinate cellular adaptation and stress responses. However, persistent ROS generation can overwhelm antioxidant defenses, disrupt redox homeostasis, and lead to mitochondrial DNA damage, protein oxidation, lipid peroxidation, and impaired bioenergetic function [4]. These events have been proposed to contribute directly to drusen formation, RPE dysfunction, and AMD progression.

Substantial evidence supports a role for mitochondrial dysfunction in AMD. Human donor tissues and cultured RPE cells demonstrate increased mitochondrial DNA damage, reduced ATP production, impaired mitophagy, altered respiratory chain activity, and progressive deterioration of mitochondrial ultrastructure [[5], [6], [7], [8], [9], [10], [11], [12]]. Yet a critical unanswered question is whether these mitochondrial alterations are associated with stress-responsive remodeling, degenerative remodeling, or different stages of the same biological process. Addressing this question has been challenging because most human studies rely on postmortem tissues with variable preservation or in vitro systems that incompletely capture the metabolic complexity of the aging retina. Likewise, rodents lack a macula and do not spontaneously develop hallmark AMD lesions such as drusen, limiting their translational relevance.

Nonhuman primates (NHPs) provide a unique opportunity to investigate mitochondrial and redox mechanisms associated with retinal aging because they possess a true macula and spontaneously develop age-related retinal lesions similar to those observed in humans. We previously characterized two distinct AMD-related phenotypes in aged rhesus macaques: soft drusen, consisting of extracellular lipid deposits between the RPE and Bruch's membrane, and punctate deposits, consisting of intracellular lipid accumulations within individual RPE cells [[13], [14], [15], [16]] (Supplemental Fig. 1). Soft drusen in NHPs are associated with age and resemble human drusen on both histology and ultrastructure [16,17]. Punctate deposits, while not a major feature of human AMD, reflect intracellular lipids similar to those observed in RPE cultured from human donors with AMD and other macular degenerations [18,19]. These phenotypes provide unique models to examine how mitochondrial remodeling and redox-associated processes differ among age-related retinal lesions in a primate species.

In this study, we investigated mitochondrial remodeling and redox-associated alterations in aged rhesus macaques with AMD-related retinal phenotypes by integrating quantitative ultrastructural analysis of RPE mitochondria with *in vivo* flavoprotein fluorescence imaging, plasma metabolomics, and whole-exome sequencing. The exceptional preservation of retinal tissues obtained immediately at necropsy enabled high-resolution assessment of mitochondrial morphology, network dynamics, and pathological remodeling states. We hypothesized that AMD-related retinal phenotypes would exhibit distinct mitochondrial ultrastructural and metabolic signatures associated with differing responses to chronic retinal stress. Our findings identify distinct mitochondrial remodeling patterns associated with punctate deposits and soft drusen, providing a framework to investigate how mitochondrial remodeling and redox homeostasis may contribute to retinal aging and AMD-related pathology.

## 2 Methods

### 2.1 Study animals & ophthalmic examinations

The CNPRC is accredited by the Association for Assessment and Accreditation of Laboratory Animal Care (AAALAC) International. All studies involving rhesus macaques (*Macaca mulatta*) adhered to the guidelines of the Association for Research in Vision and Ophthalmology Statement for the Use of Animals in Ophthalmic and Vision Research. They also complied with the NIH Guide for the Care and Use of Laboratory Animals and were approved by the UC Davis Institutional Animal Care and Use Committee. Complete ophthalmic examinations were conducted as part of a survey of eye pathologies in aged rhesus macaques (≥14 years). Animals were sedated with intramuscular ketamine hydrochloride, midazolam, and dexmedetomidine. Pupils were subsequently dilated with topical phenylephrine, tropicamide, and cyclopentolate. All animals underwent rebound tonometry, external photography, slit-lamp ophthalmoscopy, and dilated fundus biomicroscopy performed by a board-certified ophthalmologist or a veterinary ophthalmologist. Animals were classified as having soft drusen, punctate deposits, or a normal fundus, based on previously reported criteria [16]. Animals that had other retinal or choroidal lesions were excluded from the study.

### 2.2 Flavoprotein fluorescence imaging

We performed flavoprotein fluorescence (FPF) imaging in rhesus macaques using a clinically approved imaging device (OcuMet Beacon®, OcuSciences, Ann Arbor, MI) that employs an excitation band-pass filter centered at 465 nm and an emission band-pass filter to maximize retinal FPF and minimize contamination from lipofuscin and other fluorophores. Animals were positioned prone on a padded cart, with eyelids retracted using a Barraquer speculum and the cornea lubricated with artificial tears (GenTeal). We acquired 23 × 23-degree infrared and FPF images centered on the fovea, with algorithmic lens fluorescence compensation and automated selection of the highest-contrast image from multiple images captured per imaging session to ensure adequate illumination, minimal motion or blink artifacts, pupil cropping, and no registration/segmentation failures or glare. Images with a Matlab-based quality index below 30 were excluded. FPF intensity and heterogeneity were calculated using the manufacturer's proprietary software. Generalized estimating equations (GEE) with an exchangeable working correlation structure were used for group comparisons, clustering by animal to account for inter-eye correlation; model estimates represent population-averaged effects. Statistical analyses were performed using R (version 4.5.2) and RStudio (version 2025.09.2 + 418), and figures were prepared using GraphPad Prism (version 11.0.0).

### 2.3 Transmission electron microscopy

TEM analyses included samples from NHPs with normal, punctate, and drusen phenotypes, with no significant differences in age or sex distribution among groups (P = 0.371 and P = 1.000, respectively; Supplemental Table 4). Immediate preservation of ocular tissues was achieved at animal necropsy by intravitreal injection of 2% paraformaldehyde and 2.5% glutaraldehyde in 0.1 M sodium phosphate via a 25-gauge needle through the pars plana, before enucleation and immersion in the same fixative. After 48+ hours, the macular region was dissected to capture the drusen and/or punctate dot pathology. Tissues were postfixed with 2% osmium tetroxide, dehydrated with 50% to 100% ethanol, pre-infiltrated with propylene oxide and epoxy resin, and then infiltrated with resin for 24+ hours. Semithin sections were cut at 1.5 μm thickness, and ultrathin sections were cut at 100-nm thickness using a Leica EM UC6 ultramicrotome, and TEM was performed using a Talos L120c system (Thermo Fisher) and imaged with a Ceta-M 16 MP camera (Thermo Fisher). TEM images were captured at 2000× magnification to assess overall organelle identification and distribution in RPE cells at or near punctate dot lesions and at or overlying soft drusen lesions based on our prior classification motif [17], and at 13500x to focus on basolateral mitochondria sampled across the entire section. A total of 879 mitochondrial TEM images were captured, de-identified, and randomized for quantitative analysis by a separate team of 14 masked image graders.

### 2.4 Quantitative analysis of mitochondrial morphometry

Quantification analysis methods were developed using ImageJ software (NIH). From a total of 992 images, 113 were excluded due to ambiguous categorization, duplication, or low-magnification images where mitochondrial ultrastructure could not be reliably resolved (e.g., >500 nm). Among the 879 mitochondrial TEM images used for analysis, most were captured as 5 × 5 μm fields at 4096 × 4096-pixel resolution (1.07 nm/pixel, n = 673), whereas the remainder were captured at 2048 × 2048-pixel resolution (2.14 nm/pixel, n = 206), based on our previous reports on mitochondrial morphology [20,21]. All images and indicated parameters were assessed with minor modifications as detailed previously [22]. Essentially, all images were reviewed by two independent, masked, and randomly-assigned image graders to maximize rigor and minimize bias. Any measurements with a coefficient of variation (CV) > 20% between the first two graders were re-measured by a third independent image grader. When two of the three measurements showed concordance (CV ≤ 20%), the consistent measurements were averaged and used for analysis. Measurements for which consistency could not be achieved among graders (n = 65; <1%) were excluded from statistical analyses. TEM images were randomly assigned to graders' assignments, stratified by the three groups (normal, punctate deposits, drusen). Graders identified, circled, and numbered all non-cropped mitochondria in each image using Notability (Version 14.8) or Microsoft Paint (Windows 11). Image scales were manually measured from the software-annotated scale bar in ImageJ. Each uncropped mitochondrion was traced along the outer perimeter of the outer membrane using the cursor and the freehand selection tool to measure area (nm [2]), mean pixel value, minimum and maximum pixel values, perimeter, major axis, minor axis, circularity, and solidity using the ‘Area’, ‘Mean grey value’, ‘Min & max grey value’, ‘Perimeter’, ‘Shape descriptors’, and ‘Fit ellipse’ functions from the “Analyze” menu of ImageJ, and by calculating the aspect ratio, integrated density, and convex hull.

### 2.5 Evaluation of mitochondrial ultrastructural characteristics

Image graders annotated and quantified notable ultrastructural changes in each mitochondrion, including fusion or fission, megamitochondria, type I and II PCIs, and mitochondria containing concentric membranes, which may denote alterations in mitochondrial function or integrity. For each image, graders recorded the number of mitochondria exhibiting each feature, allowing calculation of feature frequencies. Based on prior studies identifying mitochondrial perimeter and solidity as key morphological parameters to predict future fission or fusion events, respectively [23], we established tolerance thresholds (two-tailed, 95% confidence intervals) from the total combined data on perimeter normalized to area (P/A), and solidity from all mitochondria regardless of phenotype, and annotated each mitochondrion as no change, at high risk for fission (P/A > upper tolerance interval) or at high risk for fusion (mitochondria with solidity > the upper tolerance interval). Megamitochondria were identified as mitochondria that appeared substantially larger than the average mitochondrial size observed in each micrograph (2 SD > mean). PCIs were categorized into type I inclusions, which displayed a well-organized, lattice-like ultrastructure, and type II inclusions that appeared less ordered. Mitochondria containing concentric membranes exhibited internal membrane structures arranged in an onion-like or circular pattern. Binary classification was applied at the individual mitochondrion level, with each ultrastructural feature graded as present or absent. For assessment of inter-grader agreement, individual binary classifications were aggregated at the image level as feature counts (i.e., the number of mitochondria per image exhibiting a given feature). Quantitative agreement between graders was evaluated using the coefficient of variation (CV) calculated from these image-level feature counts. Images with CV values > 20% were reviewed and reconciled by a third independent grader. All mitochondrial ultrastructural features were graded by two independent, masked, and randomly assigned image graders. Associations between AMD phenotype and mitochondrial morphological categories were evaluated using binomial regression models with bias reduction. For each mitochondrial morphology (e.g., hyperfused, megamitochondria with ribbon inclusions, megamitochondria without ribbon inclusions, paracrystalline inclusions, and concentric cristae), a binary outcome variable indicating the presence or absence of the morphology was modeled as a function of phenotype (Normal, Punctate, or Drusen). Because some mitochondrial morphologies were rare and could lead to separation in standard logistic regression, a bias-reduced binomial regression was implemented using Firth's penalized likelihood approach.

For each animal, the number of mitochondria exhibiting a given morphology and the total number of mitochondria evaluated were modeled using a binomial framework, thereby accounting for differences in sampling effort among animals. Thus, phenotype associations were estimated at the animal level rather than treating individual mitochondria as independent biological replicates. Odds ratios (ORs) and 95% confidence intervals (CIs) were calculated relative to the Normal phenotype as the reference category.

### 2.6 Statistical analysis for ultrastructural data

We assessed normality using multiple statistical tests (Shapiro-Wilk, Shapiro-Francia, Anderson-Darling, Kolmogorov-Smirnov, D'Agostino Skewness, D'Agostino Kurtosis, D'Agostino Omnibus, Jarque-Bera, and Q-Q plots), and transformed non-normal data using the Johnson SU method. Outliers were detected using the Cauchy robust fit algorithm with a threshold of Kσ = 4, and replaced using a low-rank approximation to preserve the underlying data structure. Missing values were imputed using multivariate robust PCA. Group differences were evaluated using permutational multivariate analysis of variance (PERMANOVA) applied to the five-dimensional feature matrix, with 999 permutations, to test for differences among control, punctate deposits, and soft drusen samples. To minimize pseudoreplication, phenotype-level inferences were based on animal-level aggregation where appropriate, whereas individual mitochondrial measurements were used to characterize ultrastructural distributions within each phenotype. Because the large number of mitochondria analyzed could yield statistically significant differences of limited biological relevance, we also computed Cohen's d (the difference in group means divided by the pooled standard deviation) to quantify effect sizes and interpreted d ≥ 0.5 as indicative of biologically meaningful differences [24].

### 2.7 Machine learning model of mitochondrial morphology

For machine learning classification and morphometric analysis, the transformed data were split into training (n = 6116) and validation (n = 2039) sets using stratified sampling across three phenotypes. The training-validation partitioning was performed at the animal level. A bootstrap forest model (JMP Pro, v. 18.2.1) was trained using 47 trees, 8 predictors sampled per split, bootstrap samples equal to the training set size, minimum split size of 8, and minimum splits per tree of 10. The top five contributors (∼80% total contribution: aspect ratio, solidity, area/perimeter ratio, minor axis, and integrated density) were used for downstream visualization. Mitochondrial morphology was analyzed using Uniform Manifold Approximation and Projection (UMAP) with two output dimensions, n_neighbors = 15, min_dist = 0.01, random seed = 123, ANNOY nearest neighbor method, stochastic gradient descent (SGD) optimizer, and angular distance metric. Morphological differences among control, punctate deposits, and soft drusen samples were tested for statistical significance using PERMANOVA on the original five-dimensional feature matrix with 999 permutations.

### 2.8 Plasma metabolomic profiling

We collected whole blood from animals maintained on a standardized diet (High Protein Monkey Diet Jumbo 5047) in the morning after at least 6 h of fasting and prior to anesthesia. Although longer fasting periods are often used in human metabolomic studies, the fasting interval was determined by rhesus macaque husbandry requirements. All animals were maintained on the same diet and sampled under identical fasting conditions. Plasma was separated and stored at −80 °C until analysis. We extracted samples using published protocols for untargeted metabolomic profiling at the West Coast Metabolomics Center (WCMC, University of California, Davis) [25,26]. Metabolite profiling by hydrophilic interaction chromatography–mass spectrometry (HILIC–MS) was conducted using an Agilent 1290 UHPLC system coupled to a Sciex TripleTOF 6600 mass spectrometer. Metabolites were separated on a Waters Acquity UPLC BEH Amide column using a binary mobile phase consisting of (A) 100% H_2_O with 10 mM ammonium formate and 0.125% formic acid and (B) 95:5 acetonitrile/H_2_O with 10 mM ammonium formate and 0.125% formic acid, with chromatographic run time of 15 min, to acquire data in data-dependent acquisition mode with scan ranges of 50–1500 *m*/*z* (MS1) and 40–1000 *m*/*z* (MS2). Primary metabolism was analyzed by gas chromatography–mass spectrometry (GC–MS) using an Agilent 7890A GC coupled to a Leco Pegasus HT TOF mass spectrometer. Dried extracts were derivatized by methoxyamination and trimethylsilylation, and injected in splitless mode with a temperature gradient from 50 to 330 °C. Mass spectra were acquired at 17 Hz over the range 85–500 Da. Data processing for LC–MS and LC–MS/MS datasets was performed using MS-Dial (version 4.9) with data-independent MS/MS deconvolution for metabolite annotation. GC–MS data were processed using ChromaTOF software and annotated against the BinBase metabolomics database. Quantification was based on peak height, which provides robust estimates of metabolite abundance with minimal influence from baseline variation. Internal standards were used for retention-time alignment and normalization, and pooled plasma quality-control samples were injected periodically to monitor analytical drift. Features with a coefficient of variation greater than 30% in QC samples were excluded from downstream analyses.

### 2.9 Statistical analysis for metabolomics data

Raw metabolite intensities were first evaluated for normality, and non-normal variables were transformed using the Johnson–Su method (JMP Pro). Missing values were imputed using robust PCA. Because mitochondrial dysfunction and redox imbalance are often better captured by relative fluxes rather than absolute metabolite abundances, we constructed a priori biologically-informed metabolite ratios representing (1) glycolytic and TCA cycle coupling (e.g., lactate/pyruvate, citrate/isocitrate, malate/fumarate), (2) redox buffering (e.g., cystine/cysteine, methionine sulfoxide/methionine), (3) fatty acid β-oxidation (e.g., 3-hydroxybutyrate/glucose), and (4) membrane and phospholipid turnover (e.g., glycerophosphatecholine/choline). Ratios were calculated only from metabolites reliably detected across samples and were tested alongside individual metabolites in the initial screening to determine whether absolute levels or flux-related indices were more discriminatory across groups. Metabolites and metabolite ratios were entered into JMP Response Screening to identify features significantly associated with each phenotype, and separate models were fit for each variable to produce FDR-corrected q-values. Only metabolites or ratios meeting an FDR threshold of q < 0.05 were used for group-wise comparison. To compare metabolite and ratio distributions across the control, punctate deposits, and soft drusen groups, we used the Nonparametric Comparison for Each Pair procedure (Wilcoxon rank-sum test). Pairwise Wilcoxon tests were FDR-corrected, and effect directions and median differences were extracted for interpretation (**Supplemental Dataset 1**). To further account for within-animal correlation and potential confounders, we also employed mixed-effects models for selected metabolites and ratios, with phenotype (control, punctate deposits, drusen) as a fixed effect and animal identity as a random effect. Models were fitted using restricted maximum likelihood (REML), and significance was assessed using FDR-adjusted p-values for fixed effects.

### 2.10 Whole exome sequencing

We prepared DNA libraries for whole-exome sequencing with the Rhexome v2 capture reagent [PMID: 31321455] on an Illumina NovaSeq 6000 [PMID: 38969634]. The reads were aligned to the rhesus Mmul_10/rheMac10 (GCF_003339765.1) reference genome assembly using BWA mem v0.7.12. To identify multiple reads that may originate from a single DNA fragment and mark them in the BAM files, we used Picard MarkDuplicates (v2.6.0; http://broadinstitute.github.io/picard/). The GATK 4.1.2.0 pipeline was used to identify SNVs following Best Practices, and a jointly called VCF file was generated. The hard filters recommended by the GATK developers (https://gatk.broadinstitute.org/hc/en-us/articles/360035532412?id=11097) were applied to the SNVs, and all variants that failed the filters were removed. Variant Effect Predictor (VEP) v108 was used to annotate variants based on merged Ensembl and RefSeq rhesus gene models. SNVs were further examined by reciprocally lifting the rhesus positions to the orthologous human positions using Picard LiftOverVcf. The functional impact of variants was predicted using CADD.

### 2.11 Statistical analysis for genetic associations

SNVs were analyzed for association with retinal phenotypes using GEMMA, which implements a linear mixed model that accounts for relatedness among individuals. The age at which the retinal phenotyping was performed was used as a covariate in the association analyses. We examined only SNVs in mitochondria or in mitochondria-associated nuclear genes, applying an FDR multiple-testing correction and a significance threshold of FDR < 0.05. Using previously published classification of drusen size and punctate dot severity [17], we compared animals with drusen (n = 35) to control animals (n = 72) based on presence of 1) any drusen, 2) medium or large drusen, or 3) large drusen only, and animals with punctate deposits (n = 101) to controls (n = 72) based on punctate dot severity of: 1) any punctate deposits, 2) moderate-severe punctate deposits, or 3) only severe punctate deposits.

### 2.12 Computational pipeline for identifying genes involved in deposit-forming disorders

To identify genes potentially involved in the pathogenesis of disorders characterized by punctate or drusen deposits, a multi-step analytical approach was employed using DisGeNET v6, STRING, and g:Profiler. Initially, gene-disease associations were extracted from DisGeNET, and only genes with a Gene-Disease Association (GDA) score of 0.5 or higher were selected, indicating a moderate-to-strong likelihood of contributing to the development of the associated condition. This threshold helped ensure that only genes with a plausible causative role were considered. After compiling the initial gene set, a targeted literature review was conducted in PubMed to validate each gene's relevance to the formation or accumulation of punctate and/or drusen deposits. Manually reviewing over 150 studies in the primary literature was essential to verify the biological importance of the chosen genes, beyond computational forecasts alone. The refined gene list was subsequently analyzed using STRING v11.5, a database and tool for evaluating known and predicted protein-protein interactions. STRING was used to assess the functional associations between genes, generating a network based on interaction confidence scores. The strength of gene-gene relationships was quantified and visualized, with the x-axis of the interaction plots representing -log (signal strength), enabling clear depiction of connection reliability and trait co-occurrence frequency.

## 3 Results

### 3.1 In vivo evidence of metabolic oxidative stress in NHPs with AMD-related lesions

**Fig. 1:**
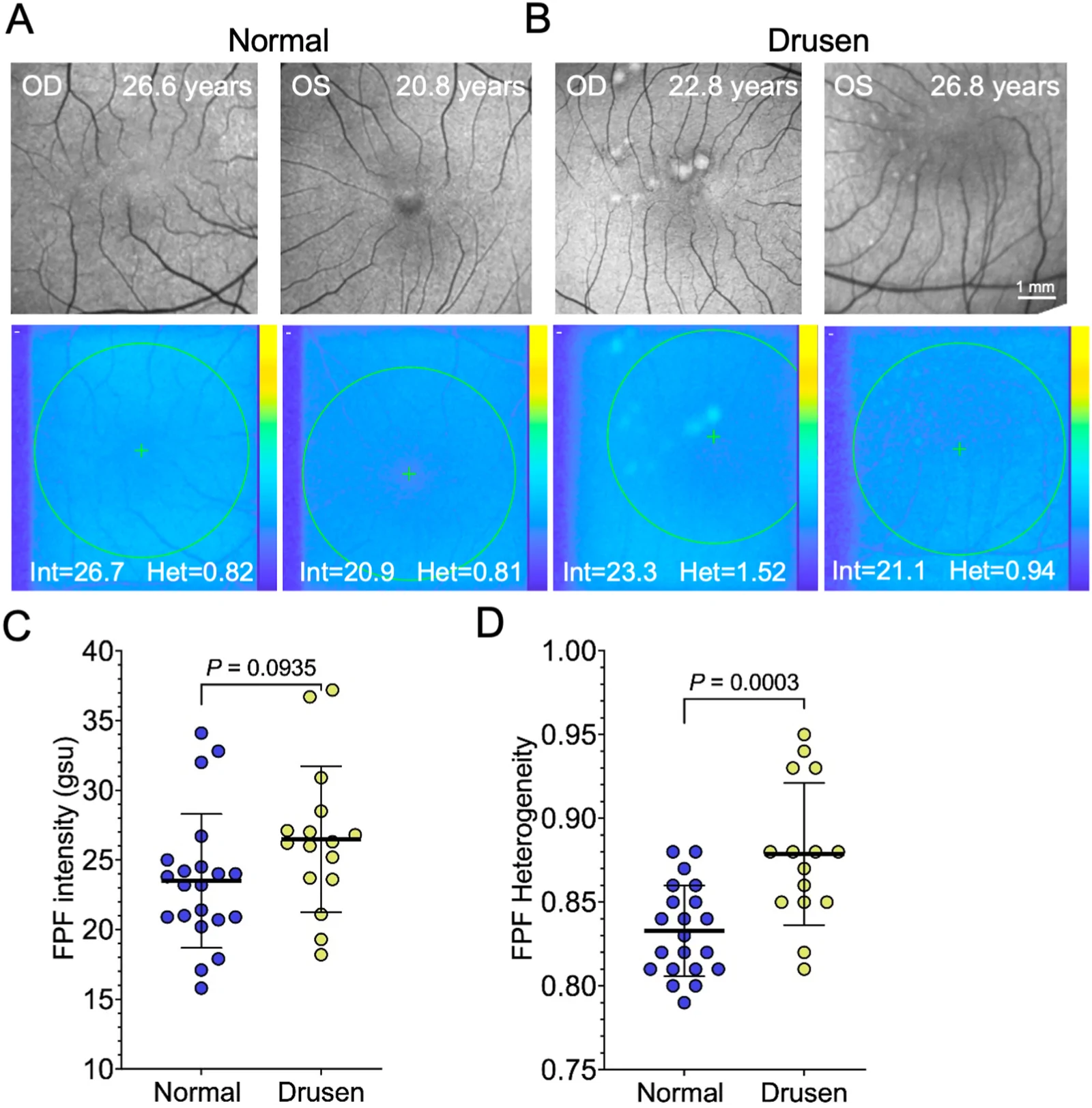
***In vivo imaging of mitochondrial redox-associated fluorescence in NHPs with soft drusen*** **(A, B)** Representative blue-peak fundus autofluorescence (FAF, top row) and fundus flavoprotein fluorescence (FPF, bottom row) of rhesus macaque eyes with (A) normal fundus and (B) soft drusen. **(C, D)** Scatterplots comparing intensity (C) and FPF heterogeneity (D) between eyes with normal fundus and soft drusen. Scale bar, 1 mm. Generalized estimating equations were used to account for inter-eye correlation in group comparisons within this age-matched cohort.

Flavoprotein fluorescence (FPF) is a noninvasive retinal imaging modality that detects an endogenous autofluorescence signal believed to arise predominantly from oxidized flavoproteins generated by mitochondrial energy production. Because flavoproteins are only fluorescent in the oxidized state, changes in FPF may serve as a biomarker of mitochondrial redox status and oxidative stress [27,28]. We previously performed macular FPF imaging in a cohort of healthy rhesus macaques and found that FPF levels increased with age, similar to human studies [28,29]. For this study, we identified 37 adult rhesus macaques (mean age 23 ± 3 years, 35 females, 2 males), which corresponds to roughly 69 human years based on a 1:3 ratio in biological aging [[30], [31], [32]], including animals with soft drusen (n = 16) and age-matched controls without retinal pathology (n = 21; Fig. 1A and B). Representative multimodal retinal imaging and histologic characterization of normal retinas, punctate dots, and soft drusen are shown in Supplementary Fig. 1. The lesions closely resemble previously described AMD-like retinal phenotypes in nonhuman primates [17].

Consistent with studies in human AMD [33], macaques with soft drusen exhibited significantly greater FPF heterogeneity (*P* = 0.0003) and a trend toward increased FPF intensity (*P* = 0.0935; Fig. 1C and D), indicating spatially heterogeneous accumulation of oxidized flavoproteins within the macula. Individual drusen lesions also displayed elevated fluorescence signals (Fig. 1B). Because FPF is thought to reflect, at least in part, the oxidized state of mitochondrial flavoproteins, these findings are consistent with localized alterations in mitochondrial redox status in eyes with soft drusen. However, FPF is an indirect imaging measure and does not, by itself, establish mitochondrial dysfunction or oxidative damage.

### 3.2 Distinct mitochondrial remodeling states emerge during aging and AMD

**Fig. 2:**
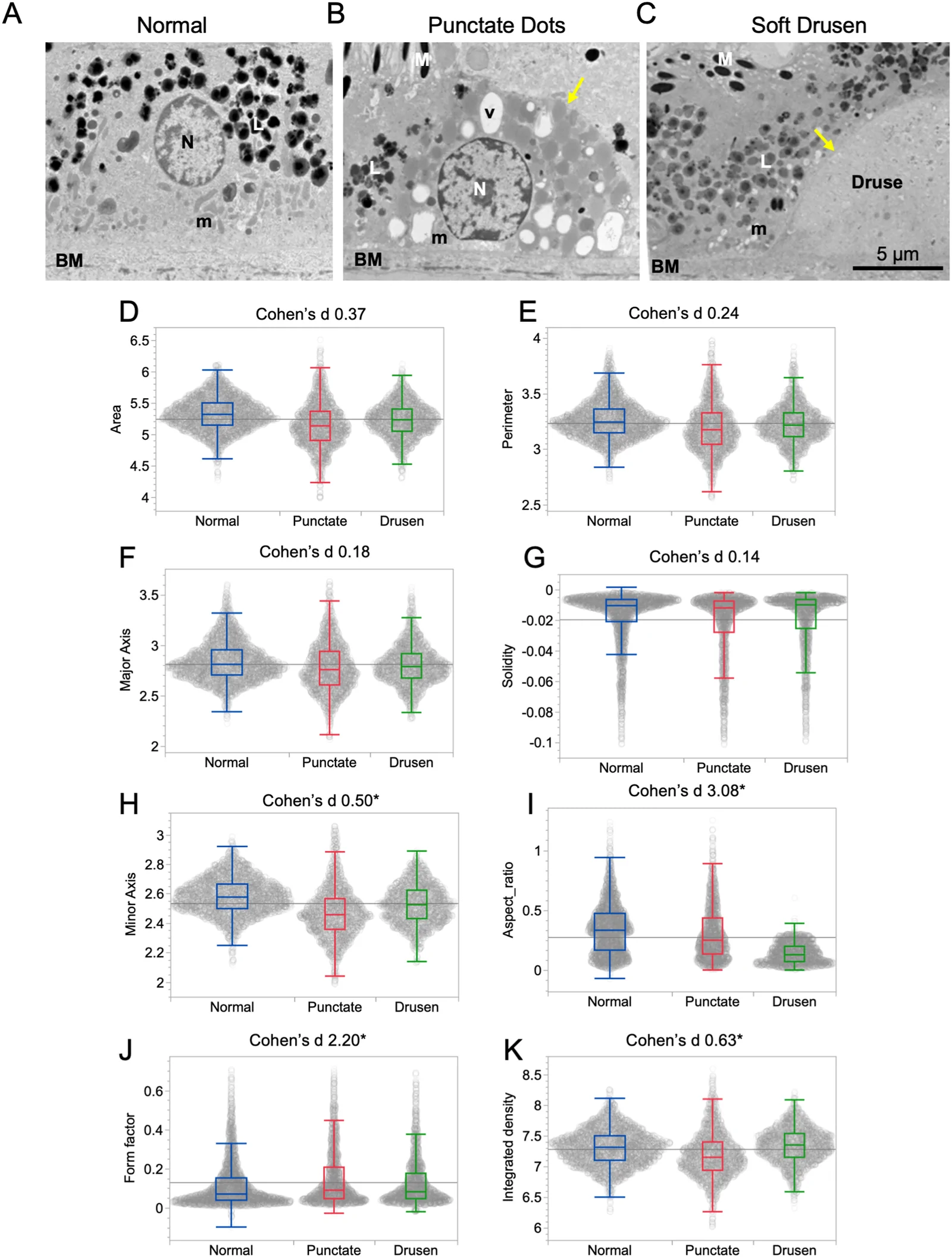
***Ultrastructural RPE mitochondrial morphometry in NHPs with AMD-related phenotypes*** **(A-C)** Representative TEM images from (**A**) normal fundus, (**B**) punctate lesions, and (**C**) soft drusen. L, lipofuscin; m, mitochondria; N, nucleus; Me, melanosomes; BM, basement membrane. (**D–K**) Violin plots showing quantitative morphometric parameters of RPE mitochondria, including (**D**) area, (**E**) perimeter, (**F**) major axis length, (**G**) solidity, (**H**) minor axis, (**I**) aspect ratio, (**J**) form factor, and (**K**) integrated density. All parameters differed significantly across the three phenotypic groups at P < 0.0001, except form factor drusen vs. punctate, P = 0.0106, and solidity drusen vs. normal, P = 0.3323; using nonparametric comparisons for each pair using the Wilcoxon method. TEM images and quantitative ultrastructural analyses from NHPs classified as normal (n = 5), punctate (n = 4), or drusen (n = 3). Individual NHP demographic characteristics are provided in Supplemental Table 4.

To investigate RPE health at the ultrastructural level, we collected TEM images from the macular region of 12 aged rhesus macaques that underwent necropsy for natural causes, including 215 images from eyes with soft drusen (n = 3, mean age 24 ± 2 years, 2 females, 1 male), 338 images from eyes with punctate deposits (n = 4, mean age 21 ± 4 years, 3 females, 1 male), and 326 images from age-matched control animals without retinal pathology (n = 5, mean age 20 ± 4 years, 4 females, 1 male; Supplemental Table 4). For the first two groups, images were collected from RPE overlying or adjacent to the AMD-related pathology. Ultrastructural analysis of macular RPE revealed most melanosomes (M) located near the apical ciliary processes, scattered lipofuscin (L) granules across the cell body, and most mitochondria (m) located near the basolateral basement membrane (BM; Fig. 2A), consistent with the distribution seen in humans [34]. Eyes with soft drusen and punctate deposits had more lipofuscin granules and smaller mitochondria than normal eyes. Mitochondria in eyes with soft drusen were also more circular or rounded, whereas those in eyes with punctate deposits appeared thinner and more elongated and irregular in shape (Fig. 2B–C).

We next performed quantitative analyses to measure (i) mitochondrial morphometry and shape complexity to quantify swelling, elongation, and fragmentation, as well as (ii) mitochondrial content/optical density as a proxy for matrix content and vacuolization, and as an indicator of potential structural compromise (Supplemental Table 1). Using robust principal component analysis (PCA) with an effect size threshold of Cohen's d ≥ 0.5 to indicate meaningful biological effects, we identified 4 morphological parameters - minor axis, aspect ratio, form factor, and integrated density - for interpretation (Fig. 2D–K). In normal aging, mitochondria exhibited the greatest minor axis, corresponding to larger mitochondrial width, and maintained an elongated morphology (Fig. 2H and I). These features are consistent with structurally intact mitochondria in healthy macaque RPE.

Mitochondria in eyes with punctate deposits displayed reduced width while maintaining an elongated morphology and exhibited the highest form factor, indicating increased branching complexity (Fig. 2J–K). These structural features are consistent with a stress-responsive remodeling pattern characterized by increased mitochondrial connectivity and structural plasticity. The lower integrated density observed in this group may reflect differences in matrix organization, although mitochondrial metabolic activity cannot be inferred directly from electron density alone. In contrast, mitochondria associated with soft drusen exhibited a rounded morphology, reduced structural complexity, and elevated integrated density (Fig. 2I–K). Together, these features are consistent with altered mitochondrial architecture and matrix organization. Our observations indicate that soft drusen are associated with a mitochondrial remodeling pattern distinct from that observed in punctate deposits and normal-aged eyes. Direct functional studies may help determine whether these structural changes correspond to impaired bioenergetic capacity or loss of mitochondrial resilience.

### 3.3 Ultrastructural mitochondrial responses associated with chronic stress

**Fig. 3:**
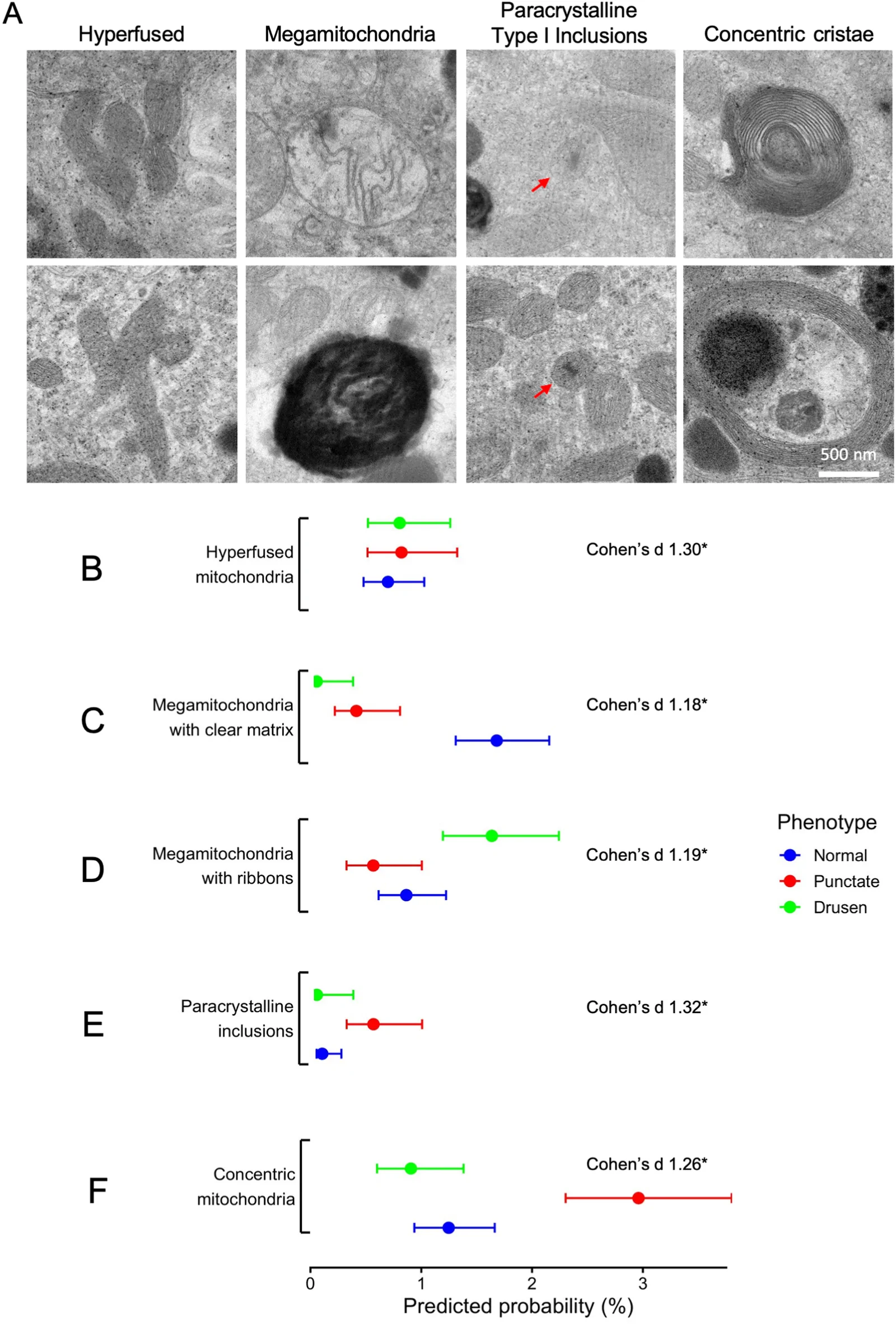
***Ultrastructural markers of RPE mitochondrial remodeling in NHPs with AMD-related phenotypes*** (**A**) Representative transmission electron microscopy (TEM) images illustrating mitochondrial morphologies associated with the different retinal phenotypes, including hyperfused mitochondria, megamitochondria with ribbon-like inclusions, type I paracrystalline inclusions, and mitochondria with concentric cristae arranged in an onion-like pattern. Scale bar, 500 nm. (**B–F**) Predicted probabilities of mitochondrial morphologies, including (**B**) hyperfused mitochondria, (**C**) megamitochondria with clear matrix content, (**D**) megamitochondria with ribbon-like inclusions, (**E**) type I paracrystalline inclusions, and (**F**) mitochondria with concentric cristae. Probabilities were estimated using bias-reduced binomial regression models that accounted for the total number of mitochondria per animal. Error bars represent 95% confidence intervals. Analysis and plot performed with RStudio. Individual NHP demographic characteristics are provided in Supplemental Table 4.

To determine whether the mitochondrial morphologies identified in AMD-related phenotypes were associated with distinct remodeling patterns, we examined ultrastructural markers of mitochondrial dynamics, including fission, fusion, hyperbranching, megamitochondria formation, paracrystalline inclusions (PCI), and concentric cristae (Fig. 3A). Although not a direct measure of mitochondrial function, these features collectively provide structural evidence of mitochondrial plasticity and stress-associated remodeling.

Mitochondrial fusion promotes network connectivity, oxidative phosphorylation efficiency, and stress resistance, whereas fission facilitates mitochondrial quality control and mitophagy but may also be associated with increased uncoupling and reactive oxygen species (ROS) production. Using previously established perimeter and solidity thresholds as proxies for fission and fusion propensity [23], we found that morphologies associated with fission remained uniformly low across phenotypes. In contrast, morphologies associated with fusion differed markedly (Chi-square test, p < 0.001), with punctate deposits occurring most frequently (13.3%), followed by soft drusen (3.1%) and normal aging (1.2%). The large effect size (Cohen's d = 1.24) indicates a substantial shift toward fusion-associated morphology in eyes with punctate deposits, consistent with increased mitochondrial connectivity under chronic retinal stress. Such remodeling may represent a compensatory response, although *in vivo* functional adaptation was not directly tested in our study.

Because fusion and fission frequencies alone do not fully capture long-term remodeling states, we next evaluated phenotype-specific ultrastructural adaptations using bias-reduced binomial regression. Hyperfusion, characterized by highly interconnected mitochondrial networks resulting from sustained fusion and reduced fission [35], was observed across all phenotypes and did not differ significantly among groups (Fig. 3B). These findings suggest that transient fusion activity, rather than stable hyperfusion, distinguishes the punctate deposit phenotype.

Megamitochondria are commonly observed under conditions of oxidative stress, bioenergetic challenge, and impaired mitochondrial turnover [36,37]. Quantitative morphometric analysis confirmed that megamitochondria were substantially larger than typical mitochondria across all measured dimensions. In aged macaque RPE, megamitochondria displayed either sparse cristae with relatively clear matrices or densely packed matrices containing ribbon-like crystalline inclusions. Megamitochondria with clear matrices, which may represent an early adaptive stage of mitochondrial remodeling, were significantly less frequent in both drusen and punctate deposit eyes relative to normal aging (Fig. 3C). In contrast, ribbon-containing megamitochondria were selectively enriched in eyes with soft drusen (OR 1.94, 95% CI 1.19–3.17; Fig. 3D), consistent with more advanced structural alteration and impaired mitochondrial homeostasis.

Paracrystalline inclusions are ordered protein aggregates that have been associated with altered respiratory chain activity and chronic mitochondrial stress. They are classified into two major types: PCI type I, comprising stacked sheets forming rectangular inclusions in the intracristae space, and PCI type II, which exhibits an electron-dense substructure and is located in the intracristae and intermembrane space [38]. Only PCI type I inclusions were observed in this study. Relative to normal aging, punctate deposits demonstrated a marked enrichment of PCI type I morphology (OR 7.58, 95% CI 1.91–30.17), whereas no enrichment was detected in drusen-associated mitochondria (Fig. 3E). The selective accumulation of PCI type I inclusions in punctate deposits is consistent with a distinct stress-associated remodeling pattern. However, because PCI formation can occur under multiple pathological and metabolic conditions, its presence alone does not necessarily establish preserved or adaptive mitochondrial function.

Mitochondria containing concentric cristae, characterized by onion-like rearrangements of the inner membrane, are widely regarded as indicators of stress-induced remodeling. Although concentric cristae were observed across all phenotypes, their prevalence was significantly increased in punctate deposits (OR 2.47, 95% CI 1.66–3.69; Fig. 3F). Together with increased fusion and PCI formation, these findings support the interpretation that punctate deposits are associated with active structural remodeling in response to chronic retinal stress.

In contrast, soft drusen lacked enrichment of these adaptive ultrastructural features and instead accumulated ribbon-containing megamitochondria indicative of advanced mitochondrial deterioration. Collectively, these findings support a model in which punctate deposits are associated with greater mitochondrial structural plasticity**,** whereas soft drusen are associated with features of structural deterioration.

### 3.4 Mitochondrial remodeling states distinguish AMD-related phenotypes

To determine whether the mitochondrial remodeling patterns identified across aging and AMD-related phenotypes were morphometrically distinguishable, we evaluated the ability of mitochondrial morphometric features to classify normal aging, punctate deposits, and soft drusen. Multiple supervised learning approaches were tested using the mitochondrial parameters identified in Fig. 2. TEM images were partitioned into training (75%) and validation (25%) datasets using stratified sampling to preserve phenotype representation. Model performance was assessed using receiver operating characteristic (ROC) analysis and classification accuracy.

**Fig. 4:**
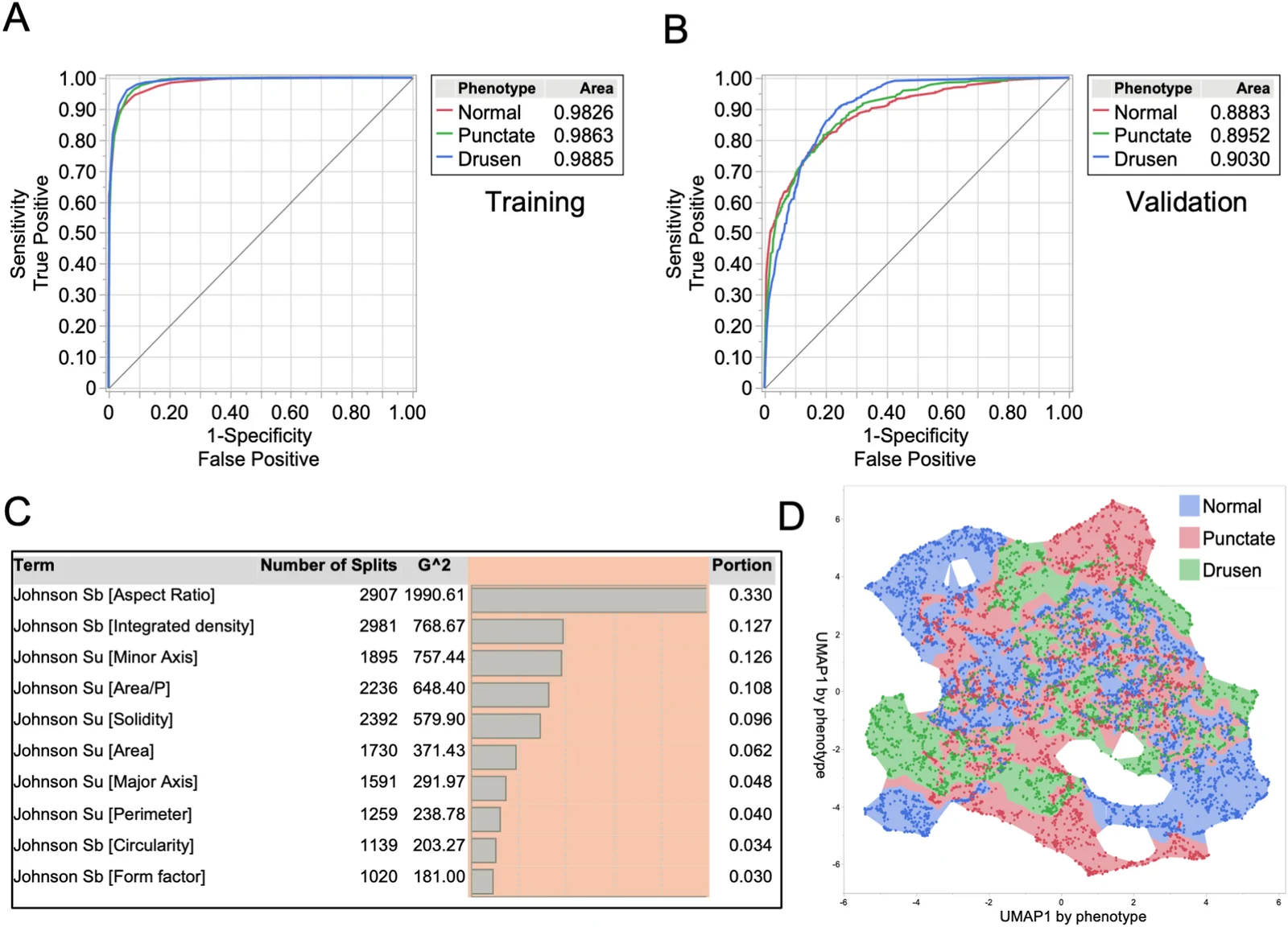
***Multivariate analysis of retinal mitochondrial morphology for phenotype classification*** (**A, B**) Receiver operating characteristic (ROC) curves for (**A**) training and (**B**) validation datasets generated from a bootstrap forest model using mitochondrial morphometric features, with corresponding area under the curve (AUC) values. (**C**) Variable importance plot showing the relative contribution of individual morphometric features to phenotype classification. Aspect ratio, integrated density, minor axis length, solidity, and area collectively accounted for approximately 80% of the model's predictive performance. (**D**) Uniform Manifold Approximation and Projection (UMAP) embedding of the top five mitochondrial morphometric features from eyes with normal fundus (blue), punctate lesions (red), and soft drusen (green). Each point represents a single mitochondrion, and density contours indicate regions enriched for a given phenotype. Although partial overlap is present among groups, phenotype-enriched regions are apparent despite partial overlap among groups, consistent with phenotype-associated differences in mitochondrial morphology. Permutational multivariate analysis of variance (PERMANOVA) performed on the original five-dimensional feature matrix confirmed significant differences among groups (pseudo-F = 341.95, P = 0.001; n = 8168 mitochondria). Individual NHP demographic characteristics are provided in Supplemental Table 4.

Among the models evaluated, the bootstrap forest classifier demonstrated the strongest performance, achieving ROC values greater than 0.980 for all phenotypes in the training dataset and greater than 0.880 in the validation dataset (Fig. 4A and B). Misclassification rates were 8.6% and 26.7% for the training and validation datasets, respectively, suggesting resasonble distinction between the retinal phenotypes, despite lower classification performance in the validation subset.

Analysis of variable importance revealed that aspect ratio was the strongest contributor to classification performance, followed by minor axis, integrated density, area-to-perimeter ratio, and solidity (Fig. 4C). Together, these five parameters accounted for approximately 80% of the model's predictive capacity, with integrated density, minor axis, and aspect ratio exhibiting the largest biological effect sizes (Cohen's d ≥ 0.5). Notably, these features correspond to mitochondrial elongation, structural complexity, and matrix organization, suggesting that alterations in mitochondrial architecture are important contributors to phenotypic classification.

To visualize relationships among mitochondrial remodeling states, we performed Uniform Manifold Approximation and Projection (UMAP) using the five highest-ranking morphometric features. Although mitochondria from all phenotypes occupied a partially overlapping continuum, phenotype-enriched regions appeared distinct (pseudo-F = 341.95, p = 0.001; Fig. 4D). Rather than forming discrete clusters, mitochondria are distributed along a continuous morphological landscape, suggesting overlapping but phenotype-associated patterns of mitochondrial remodeling.

Our findings demonstrate that mitochondrial ultrastructure captures biologically meaningful information that can differentiate AMD-related phenotypes. The ability of a small set of morphometric parameters to classify retinal lesions with moderate validation error suggests that punctate deposits and soft drusen may be associated with differing mitochondrial remodeling patterns.

### 3.5 Systemic metabolomic signatures reveal divergent redox states

Metabolomic profiling in a cohort of NHPs identified systemic metabolic differences among the retinal phenotypes. Plasma metabolomic studies in AMD patients have reported enrichment of glycerophospholipid pathways associated with mitochondrial fatty acid oxidation and membrane remodeling [[39], [40], [41]]. To determine whether AMD-related phenotypes in nonhuman primates exhibit similar metabolic signatures, we analyzed a targeted panel of 22 metabolites and biologically informed metabolite ratios selected to interrogate pathways involved in mitochondrial bioenergetics, redox homeostasis, lipid remodeling, and cellular stress responses.

The panel included markers associated with cytosolic and mitochondrial redox balance (lactate/pyruvate, methionine sulfoxide/methionine, cystine/cysteine), tricarboxylic acid (TCA) cycle metabolism (citrate/isocitrate, malate/fumarate), fatty acid oxidation and ketone metabolism, membrane phospholipid turnover (glycerophosphocholine/choline), sphingolipid signaling (sphinganine-1-phosphate), and tryptophan metabolism. Metabolite ratios were included as surrogate measures of pathway activity, as they can be more sensitive than absolute metabolite concentrations, although circulating ratios may not directly reflect metabolic flux within the retinal pigment epithelium.

Fasting plasma was collected from aged rhesus macaques with soft drusen, punctate deposits, and age-matched controls. The metabolomics study included 122 NHP participants: 75 with normal fundus appearance, 25 with drusen, and 22 with punctate dots. The mean age differed significantly among the three groups (Normal: 18.50 ± 2.72 years; Drusen: 20.47 ± 2.77 years; Punctate dots: 19.37 ± 2.69 years; one-way ANOVA, *P* = 0.008). The proportion of female participants was similar across groups (86.7%, 92.0%, and 86.4%, respectively; Fisher's exact test, *P* = 0.913). Following normalization, imputation, and mixed-effects modeling, 13 of 22 candidate metabolites and ratios were significantly associated with AMD-related phenotypes after false-discovery correction (Supplemental Table 2; Supplemental **Dataset 1**). Both lesion phenotypes exhibited elevated levels of docosahexaenoic acid, docosatetraenoic acid, linoleic acid, and glycerophosphocholine/choline ratios relative to controls, all of which are associated with phospholipid remodeling and membrane turnover. However, the two lesion types diverged markedly in their metabolic signatures. Animals with punctate deposits showed elevated levels of lactate/pyruvate, citrate/isocitrate, malate/fumarate, and sphinganine-1-phosphate, consistent with differences in glycolytic, TCA-cycle, and stress-responsive lipid pathways that parallel the increased local mitochondrial remodeling observed ultrastructurally in the RPE.

Notably, punctate deposits were also associated with reduced cystine/cysteine and 2-hydroxybutyrate levels, suggesting altered thiol-associated and glutathione-linked metabolism. Together with the enhanced fusion, paracrystalline inclusions, and concentric cristae identified ultrastructurally, these findings are consistent with a systemic metabolic profile accompanying active mitochondrial remodeling. In contrast, animals with soft drusen exhibited elevated (3-hydroxybutyrate + acetoacetate)/glucose ratios along with reduced methionine sulfoxide/methionine and 2-hydroxybutyrate levels, which may reflect differences in glucose utilization, ketone metabolism, or systemic substrate availability. The concomitant reduction in methionine- and thiol-associated metabolites is also consistent with altered systemic redox-related metabolism.

Together, the circulating metabolic abnormalities we observed paralleled the structural alterations seen in drusen-associated RPE, although a direct relationship between plasma metabolites and local RPE mitochondrial function cannot be definitively established from these data. Our findings indicate that AMD-related phenotypes in rhesus macaques are associated with distinct systemic metabolic signatures involving mitochondrial flux, lipid remodeling, and redox homeostasis. These metabolic differences show qualitative correspondence with the ultrastructural mitochondrial phenotypes observed in the RPE, and suggest that punctate deposits and soft drusen are associated with distinct systemic metabolic profiles.

### 3.6 Genetic associations involving mitochondrial respiratory function and retinal maintenance

To determine whether AMD-related phenotypes in rhesus macaques are associated with genetic variation affecting mitochondrial function, we analyzed whole-exome sequencing data using Genome-wide Efficient Mixed Model Association (GEMMA). The analysis included animals with soft drusen (n = 32), punctate deposits (n = 98), and age-matched controls (n = 72), with age at retinal phenotyping included as a covariate.

Among mitochondrial genes, we identified a significant single-nucleotide variant (SNV), MT:9582G > A (FDR = 0.012), associated with large soft drusen. This missense variant (V129 M) is in the mitochondrial cytochrome *c* oxidase subunit III (*COX3*) gene, which encodes a core component of Complex IV of the electron transport chain. The corresponding human ortholog, MT:9591G > A (V129I), is rare, with a reported allele frequency of 0.08% in the MITOMAP database. Given the central role of Complex IV in oxidative phosphorylation and mitochondrial redox balance, this finding identifies a potential genetic association with the drusen phenotype.

Analysis of mitochondria-associated nuclear genes identified four SNVs within prominin-1 (*PROM1*) associated with moderate-to-severe punctate deposits (FDR = 0.025 for all variants), including three missense variants and one synonymous variant (Supplemental Table 3). Although all variants exhibited relatively low predicted functional impact (CADD < 12), their collective association identifies *PROM1* as a candidate locus for future investigation of photoreceptor–RPE homeostasis in the punctate deposit phenotype. These findings provide complementary genetic associations linking AMD-related phenotypes in rhesus macaques to genes involved in mitochondrial respiration and retinal maintenance, providing biological context for our ultrastructural observations.

### 3.7 Exploratory pathway analyses identify phenotype-associated biological processes

To place the mitochondrial, metabolomic, and genetic findings into a broader biological context, we performed a hypothesis-generating pathway analysis using genes previously associated with drusen or punctate deposits in human studies. Gene candidates were identified through DisGeNET, g:Profiler, STRINGdb, and manual PubMed curation. Because the term “punctate deposits” in human studies may encompass heterogeneous retinal lesions, including small drusen, reticular pseudodrusen, and other punctate retinal abnormalities, these analyses were intended to provide biological context rather than establish direct equivalence with the nonhuman primate phenotypes.

**Fig. 5:**
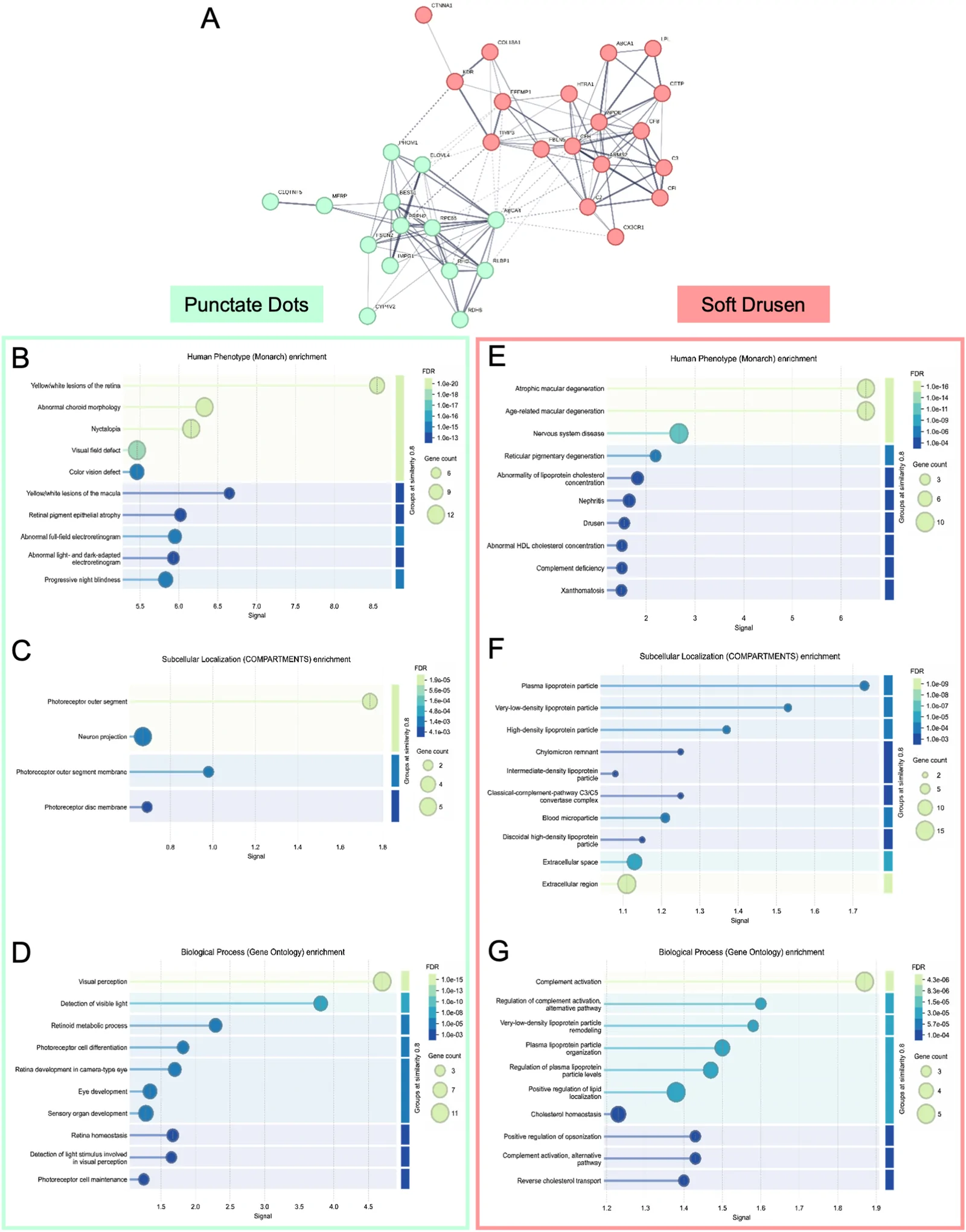
***Exploratory pathway analysis of genes associated with AMD-related retinal phenotypes*** **(A)** Gene interaction networks constructed using STRING based on 32 genes associated with “punctate” or “drusen”, obtained by integrating g:Profiler, DisGeNET, and PubMed literature mining with *k*-nearest neighbors (kNN) clustering, showing gene clusters enriched for punctate- and drusen-associated genes (green; left panels) and drusen (red; right panels) lesions. Bubble plots displaying enriched human phenotype ontology terms based on Monarch data (**B, E**), predicted subcellular localization (**C, F**), and enriched biological processes (gene ontology; **D, G**) for punctate (**B, C, D**) and drusen (**E, F, G**).

Functional enrichment analysis revealed distinct pathway signatures associated with each lesion category. Genes associated with drusen were enriched for pathways involved in cholesterol transport and complement activation, two processes strongly implicated in AMD pathogenesis and chronic retinal inflammation (Fig. 5). In contrast, genes associated with punctate deposits were more strongly enriched for pathways related to photoreceptor outer segment maintenance, retinoid metabolism, and visual cycle function.

Although these analyses do not establish causal relationships, the pathway enrichments provide broader biological context for the lesion categories examined. Drusen-associated pathways were predominantly linked to lipid accumulation and immune activation, whereas punctate-deposit-associated pathways were more closely associated with retinal maintenance and cellular homeostasis. Because these analyses rely on previously reported gene–disease associations and literature curation, they are not independent mechanistic evidence for the ultrastructural or metabolomic findings. Rather, they enable hypothesis generation on the biological processes that may differ between lesion phenotypes.

## 4 Discussion

Human studies and experimental models have implicated mitochondrial dysfunction in the pathogenesis of age-related macular degeneration (AMD), yet the relationship between mitochondrial remodeling, redox homeostasis, and disease-associated retinal lesions remains poorly understood. In this study, we integrated quantitative mitochondrial ultrastructural analysis, *in vivo* flavoprotein fluorescence imaging, plasma metabolomics, and genetic analyses in aged rhesus macaques to characterize mitochondrial alterations associated with two AMD-related retinal phenotypes. Eyes with punctate deposits exhibited increased mitochondrial fusion, and enrichment of type I paracrystalline inclusions and concentric cristae in RPE, with systemic metabolic signatures consistent with an active stress response. In contrast, eyes with soft drusen showed ultrastructural features of RPE mitochondrial deterioration, systemic metabolic changes suggesting altered substrate utilization and redox homeostasis, and genetic association with a mitochondrial respiratory-chain gene *COX3*. Collectively, these findings indicate that AMD-related lesions in nonhuman primates show divergent patterns of mitochondrial remodeling and redox-related metabolism rather than a uniform response to retinal aging. Because direct functional measurements of mitochondrial activity were not performed, however, the terms “adaptive” and “degenerative” refer to interpretations of the observed structural and metabolic patterns rather than experimentally validated functional states.

**Fig. 6:**
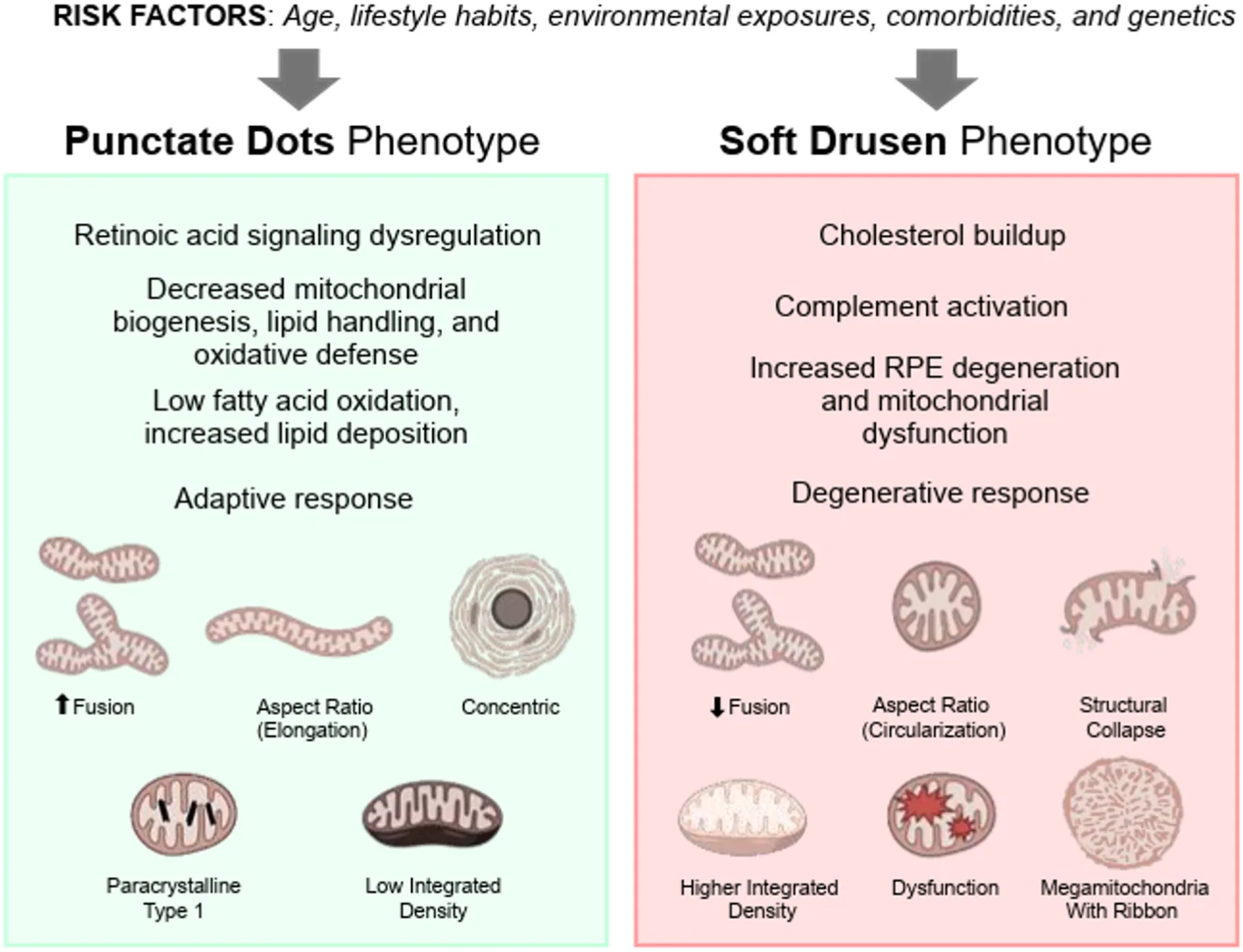
***Proposed model of mitochondrial remodeling associated with AMD-related phenotypes in NHPs*** Schematic summarizing the mitochondrial ultrastructural patterns and associated systemic metabolic signatures observed in punctate deposits and soft drusen. The model is intended as a conceptual framework derived from the ultrastructural, imaging, metabolomic, and genetic findings and does not imply direct demonstration of mitochondrial function or causal mechanisms.

A central finding of this study is that punctate deposits and soft drusen are associated with different patterns of mitochondrial remodeling. Mitochondria continuously undergo fusion, fission, biogenesis, and turnover to maintain bioenergetic function and cellular homeostasis [42,43]. These processes are particularly important in the RPE, which experiences lifelong exposure to high oxygen tension, intense metabolic demand, and chronic oxidative stress [4]. In eyes with punctate deposits, increased fusion frequency, enrichment of type I paracrystalline inclusions, and increased prevalence of concentric cristae implicate a stress-responsive remodeling pattern in which mitochondrial structural plasticity may be retained under chronic stress. By contrast, eyes with soft drusen lacked enrichment for these remodeling features and instead accumulated ribbon-containing megamitochondria, which, in other tissues, are associated with structural deterioration and impaired mitochondrial homeostasis. Our data do not establish whether these phenotypes represent distinct biological trajectories, sequential stages of a shared aging process, or different manifestations of retinal stress. Also, while soft drusen are characteristic of AMD, punctate deposits are distinct from small drusen or reticular pseudodrusen observed in human disease, and may reflect a pattern more unique to NHP's macular physiology. Nevertheless, our study revealed two retinal phenotypes with distinct ultrastructural responses: punctate deposits exhibit features of active mitochondrial remodeling, whereas soft drusen demonstrate signs of structural decline (Fig. 6).

Past studies of human AMD tissues have primarily relied on qualitative assessments of mitochondrial ultrastructure, with relatively few quantitative morphometric analyses due to inconsistent tissue preservation and variability in postmortem collection procedures [7,10,12,44]. By contrast, the present study used macular tissues preserved immediately at necropsy, enabling detailed quantification of mitochondrial morphology and ultrastructural pathology. Several of the features identified in our study have been reported previously in mitochondrial myopathies, aging tissues, and degenerative diseases. For example, megamitochondria containing crystalline inclusions have been described in nonalcoholic fatty liver disease, cardiomyopathy, and aging tissues, where they are associated with altered cristae architecture, proteostatic stress, and impaired mitochondrial quality control [35,[45], [46], [47], [48], [49]]. Type I paracrystalline inclusions have also been reported more frequently in tissues with impaired cytochrome *c* oxidase activity [35]. The prevalence of these structures in normal-aged macaques was similar to that reported in skeletal muscle biopsies from aged individuals [38]. These similarities support the relevance of the rhesus macaque for comparative studies of mitochondrial aging, although they do not establish that the functional consequences of these structures are identical across tissues or species. Future studies using similarly well-preserved human retinal tissue and direct functional measurements will be necessary to clarify the role of mitochondrial remodeling in AMD progression.

The plasma metabolomic findings provided indirect, but complementary evidence of differing systemic metabolic profiles. Both lesion groups exhibited changes in phospholipid-related metabolites and polyunsaturated fatty acids, consistent with shared disturbances in lipid homeostasis that have also been reported in human AMD metabolomic studies [39,41]. Animals with punctate deposits, however, exhibited higher lactate/pyruvate, citrate/isocitrate, malate/fumarate, and sphinganine-1-phosphate values, together with altered thiol-associated metabolites, consistent with differences in glycolytic and tricarboxylic acid cycle intermediates, lipid signaling, and redox-buffering pathways. In contrast, animals with soft drusen exhibited higher ketone-body-to-glucose ratios and lower methionine sulfoxide/methionine ratios and 2-hydroxybutyrate levels, suggesting differences in substrate utilization and systemic redox-related metabolism. These metabolomic findings should not be interpreted as direct measurements of mitochondrial activity in the RPE. Circulating metabolites can be influenced by systemic physiology, age, diet, and metabolism across multiple tissues, and therefore cannot be used to establish the functional state of RPE mitochondria. Although the animals were maintained under standardized dietary and sampling conditions and were age-matched where possible, residual systemic influences cannot be excluded. The plasma data are therefore presented as complementary associations that parallel some ultrastructural findings rather than as independent proof of retinal mitochondrial adaptation or degeneration. Future metabolomic analysis of freshly isolated RPE–choroid tissue could provide a more direct assessment of local retinal metabolism.

Our study also identified additional genetic associations that may inform future mechanistic studies. We identified a mitochondrial DNA variant in cytochrome *c* oxidase subunit III (*COX3*) associated with large soft drusen. *COX3* encodes a component of Complex IV, the terminal enzyme of the electron transport chain that contributes to oxidative phosphorylation and mitochondrial redox balance. Although this link is biologically relevant, the functional consequences of the identified variant remain unknown and were not tested in the present study. We also identified variants in *PROM1* associated with punctate deposits. *PROM1* plays a critical role in photoreceptor outer-segment morphogenesis and maintenance [50], and pathogenic variants can cause inherited macular dystrophies and bull's-eye maculopathy [51]. While the variants identified here are predicted to have modest functional effects, they suggest potential differences in photoreceptor-RPE homeostasis between lesion phenotypes.

Similarly, the pathway analyses provide additional biological context. Genes associated with drusen were enriched for cholesterol transport and complement activation pathways [52,53], which are well-established contributors to drusen formation, AMD pathogenesis, and chronic retinal inflammation. Genes associated with punctate deposits were enriched for pathways involving retinoid metabolism and photoreceptor maintenance. Because these analyses were based partly on previously-reported gene–disease associations and literature curation, they are not independent experimental evidence linking the ultrastructural phenotypes to specific molecular mechanisms, but rather implicate broader biological processes that should be considered exploratory at this time.

The role of mitochondrial dysfunction in AMD remains complex. Chronic oxidative stress in aging RPE promotes lipid peroxidation, protein modification, DNA damage, and mitochondrial dysfunction. Mitochondrial injury may impair fatty-acid oxidation, contribute to lipid accumulation in drusen, promote Slug-mediated epithelial–mesenchymal transition of RPE cells [54], and activate innate immune signaling through the cGAS–STING pathway in response to damaged mitochondrial DNA [55,56]. Conversely, AMD-associated processes may further exacerbate mitochondrial dysfunction. Chronic inflammation and complement activation can increase reactive oxygen species production, whereas drusen-associated factors may impair autophagy and mitophagy. RPE cells lacking complement factor H or expressing the AMD-associated Y402H risk variant exhibit enlarged mitochondria, reduced mitochondrial activity, and increased photoreceptor toxicity [[57], [58], [59]]. These observations support a model in which mitochondrial dysfunction may participate in a self-reinforcing cycle that contributes to retinal degeneration. Within this framework, the distinct remodeling patterns observed in the present study suggest that the capacity to maintain mitochondrial structural plasticity may influence retinal resilience during aging. Direct functional and longitudinal studies will be required to test this hypothesis.

Our findings provide a framework for future studies to determine how mitochondrial remodeling contributes to retinal aging and AMD-related pathology in nonhuman primates. Therapeutic strategies aimed at preserving mitochondrial integrity, enhancing redox homeostasis, improving mitophagy, or supporting respiratory-chain function are being investigated for retinal degenerative diseases. These include cardiolipin-binding peptides that stabilize mitochondrial cristae [60] and photobiomodulation approaches intended to influence Complex IV activity [61]. Rhesus macaques possess a true macula and spontaneously develop age-associated retinal lesions, including soft drusen lesions [13,14,16], and may provide a valuable NHP model for investigating mitochondrial remodeling and potential testing of these mitochondria-targeted therapies.

Several limitations should be considered. Our analyses focused primarily on the basal mitochondrial population of the RPE and did not evaluate subapical mitochondrial populations that may perform specialized functions, including support for outer-segment phagocytosis [62,63]. Sample sizes were constrained by the limited availability of aged rhesus macaques and the high cost of nonhuman primate studies. Consequently, some analyses may have been underpowered to detect modest effect sizes, particularly in the genetic association studies and subgroup comparisons. In addition, the imaging, metabolomic, and genetic findings are largely correlative and do not establish mechanistic relationships. Primary NHP RPE culture systems are not yet sufficiently developed to permit detailed mechanistic studies of the pathways identified here. Finally, direct functional measurements of mitochondrial respiration, oxidative phosphorylation, ATP production, oxidative damage, or mitophagy were not feasible because ocular tissues were fixed immediately after collection to preserve ultrastructural integrity. Consequently, our interpretation of adaptive and degenerative remodeling is based on convergent evidence from ultrastructural, metabolomic, imaging, and genetic analyses rather than direct functional measurements. Despite these limitations, the present work demonstrates the value of quantitative mitochondrial morphometry in exceptionally well-preserved primate retinal tissue and integrates complementary imaging, metabolomic, and genetic datasets to provide convergent evidence for mitochondrial dysfunction in AMD-related phenotypes.

## 5 Conclusion

In summary, this study identifies distinct mitochondrial-redox states associated with AMD-related phenotypes in aged rhesus macaques. Quantitative ultrastructural, imaging, metabolomic, and genetic analyses identified distinct mitochondrial remodeling patterns associated with the two retinal phenotypes. Punctate deposits are characterized by adaptive mitochondrial remodeling and metabolomic signatures consistent with active stress engagement, whereas soft drusen are associated with impaired redox homeostasis and mitochondrial degeneration. These findings support the concept that AMD-related lesions are associated with divergent mitochondrial responses to chronic stress rather than a uniform aging process. By establishing links between mitochondrial structure, metabolic state, and retinal pathology in a nonhuman primate model, this work provides new insight into the role of mitochondrial dysfunction in AMD and highlights mitochondrial remodeling and redox resilience as potential therapeutic targets.

## CRediT authorship contribution statement

**Cecilia Giulivi:** Conceptualization, Data curation, Formal analysis, Investigation, Methodology, Resources, Supervision, Validation, Visualization, Writing – original draft. **Carol Villafuerte-Trisolini:** Data curation, Formal analysis, Investigation, Methodology. **Zoe Lee Greenblatt:** Data curation, Formal analysis, Investigation, Methodology. **Jennifer Dang:** Data curation, Formal analysis, Investigation, Methodology, Validation. **Atena Tork:** Data curation, Formal analysis, Investigation, Validation. **Rida Mullah:** Data curation, Formal analysis, Investigation, Supervision, Validation, Writing – review & editing. **Sophie Le:** Data curation, Methodology, Validation. **Angela Min:** Data curation, Formal analysis, Investigation, Methodology, Supervision, Validation, Writing – review & editing. **Maya Nair:** Data curation, Formal analysis, Methodology, Supervision, Validation, Writing – review & editing. **Aldo Vorkapich:** Data curation, Formal analysis, Investigation, Methodology, Validation, Writing – review & editing. **Huy-An Tran:** Investigation, Methodology. **Elizabeth Doyle:** Formal analysis, Investigation, Methodology, Validation, Visualization. **Alestor Law:** Data curation, Formal analysis, Investigation, Methodology. **Owen O'Keefe:** Data curation, Formal analysis, Investigation, Methodology. **Annabelle Wu:** Data curation, Formal analysis, Investigation, Methodology, Supervision, Validation. **Bradley Shibata:** Data curation, Investigation, Methodology, Resources, Visualization. **Ana Ripolles-Garcia:** Data curation, Formal analysis, Investigation, Methodology. **Sara M. Thomasy:** Data curation, Formal analysis, Funding acquisition, Investigation, Methodology, Supervision, Validation, Writing – review & editing. **R. Alan Harris:** Data curation, Formal analysis, Investigation, Methodology, Validation. **Rui Chen:** Formal analysis, Investigation, Methodology. **Yin Allison Liu:** Data curation, Investigation, Methodology, Resources, Writing – review & editing. **Glenn Yiu:** Conceptualization, Data curation, Funding acquisition, Investigation, Methodology, Project administration, Resources, Supervision, Writing – review & editing.

## Ethics declaration

This study was conducted in accordance with the ARRIVE (Animal Research: Reporting of In Vivo Experiments) guidelines. This study was approved by the University of California, Davis.

## AI declaration statement

The authors used ChatGPT (OpenAI) solely for the initial generation of a graphical abstract based on an author-developed sketch and author-provided scientific concepts. The graphical abstract was subsequently modified, edited, and finalized by the authors using BioRender and Preview. No AI tools were used for data analysis, interpretation of results, or generation of scientific conclusions. The authors reviewed and approved the final graphical abstract and assume full responsibility for its content.

## Declaration of competing interest

CG received compensation from Frontiers in Molecular Biosciences and NIH. GY received research support from Abbvie and Cirrus Therapeutics, and personal fees for consultancy from 4DMT, AbbVie, Adverum, Alcon, Bausch & Lomb, Boehringer Ingelheim, Clearside Biomedical, Coave, Genentech, jCyte, Merck, Mobius, Nanoscope, Ocuphire, Opthea, Osanni, Ray BioTherapeutics, RegenXBio, Roche, Samsung Bioepis, Stealth Therapeutics, SunRegen, Supra Medical, Surrozen, and West Pharmaceutical. The remaining authors declared no conflicts of interest.

## Data Availability

The metabolomics datasets generated during this study are publicly available through the Dryad Digital Repository (https://doi.org/10.5061/dryad.7pvmcvf8d). The quantitative data supporting the analyses presented in this manuscript are included in the article and its Supplementary Information files. Due to their large size, the complete set of 879 transmission electron microscopy images is not publicly deposited but is available from Dr. Glenn Yiu upon reasonable request.
