# Supplementary material for: Adaptive and degenerative mitochondrial remodeling define distinct redox states in age-related macular degeneration
Source: Redox Biol. 2026 Aug 22;96:104365. doi: 10.1016/j.redox.2026.104365 (PMC13543981; doi:10.1016/j.redox.2026.104365)
Supplement: Supplementary file 1 [file mmc1.docx]

***Supplemental Table 1.	Mitochondrial Remodeling Patterns Indicate Separate Stress Pathways in AMD***

This table summarizes qualitative and quantitative patterns of mitochondrial remodeling in normal (N), punctate (P), and drusen-associated (D) retinal pigment epithelium. Features reflect adaptive versus degenerative mitochondrial stress responses, highlighting distinct mitochondrial phenotypes associated with disease progression in age-related macular degeneration (AMD).

| Mitochondrial Feature | N (Normal) | P  (Punctate) | D (Drusen) |
| --- | --- | --- | --- |
| **Morphologies associated with fusion** | Low | **High** | Low |
| **Hyperbranching/hyperfused** | Same | Same | Same |
| **Megamitochondria with clear content** | High | **Low** | Low |
| **Megamitochondria with ribbon-like inclusions** | Low | Low | High |
| **Paracrystalline inclusions** | Low | High | Low |
| **Concentric cristae-containing mitochondria** | Low | High | Low |
| **Integrated density** | Moderate | **Low** | **High** |
| **Aspect ratio** | High | **High** | **Low** |

***Supplemental Table 2.	Top metabolite signatures of mitochondrial dysfunction and redox imbalance in drusen and punctate lesions***

This table lists the most significantly altered metabolites and metabolite ratios associated with mitochondrial dysfunction and oxidative stress in drusen and punctate lesions. Metabolites were selected from **Supplemental Dataset 1** based on statistical significance (*P* < 0.05). Abbreviations: BCAA, branched-chain amino acids.

*Metabolites or ratios included in the above table were selected from **Supplemental Table 1** with *P* values <0.05. Abbreviations: BCAA, branched-chain amino acids.

***Supplemental Table 3.	Mitochondrial genes associated with AMD-related phenotypes in NHPs***

| SNV_ID | Gene | Consequence | CADD | Allele1 | Allele0 | AF | β | SE | P(LRT) | FDR |
| --- | --- | --- | --- | --- | --- | --- | --- | --- | --- | --- |
| MT:9582G>A | *COX3* | Missense (V129M) | NA | A | G | 0.065 | 0.182 | 0.050 | 8.77×10⁻⁴ | 0.0126 |
| 5:15032864G>A | *PROM1* | Synonymous | 11.02 | A | G | 0.167 | -0.227 | 0.072 | 1.20×10⁻³ | 0.0253 |
| 5:15008469C>T | *PROM1* | Missense | 4.14 | T | C | 0.172 | -0.219 | 0.072 | 1.52×10⁻³ | 0.0253 |
| 5:14993178T>G | *PROM1* | Missense | 0.82 | G | T | 0.183 | -0.236 | 0.071 | 6.30×10⁻⁴ | 0.0253 |
| 5:15008499G>C | *PROM1* | Missense | 0.06 | C | G | 0.172 | -0.219 | 0.072 | 1.52×10⁻³ | 0.0253 |

*******CADD scores were unavailable for the mtDNA variants and are therefore reported as NA. All *PROM1* variants exhibited low predicted functional impact (CADD < 12).

***Supplemental Table 4.	Characteristics of non-human primates included in the study related to mitochondrial morphology analyses****

| NHP ID | Phenotype | Age (years) | Sex |
| --- | --- | --- | --- |
| 33426 | Drusen | 22.38 | F |
| 26691 | Drusen | 26.19 | M |
| 32191 | Drusen | 24.27 | F |
| 31649 | Normal | 27.16 | F |
| 32237 | Normal | 18.79 | F |
| 33882 | Normal | 21.61 | F |
| 34176 | Normal | 16.69 | F |
| 36454 | Normal | 17.97 | M |
| 34453 | Punctate | 24.07 | F |
| 27750 | Punctate | 25.06 | F |
| 35726 | Punctate | 18.95 | M |
| 37341 | Punctate | 16.67 | F |
|  |  |  |  |
| Group summary |  |  |  |
| Drusen (n = 3) |  | **24 ± 2** | 1 M / 2 F |
| Normal (n = 5) |  | **20 ± 4** | 1 M / 4 F |
| Punctate (n = 4) |  | **21 ± 4** | 1 M / 3 F |
| P value |  | **0.393** | **1.000** |

*Individual data are shown together with group summaries presented as mean ± SD. Differences in age among phenotype groups using the Kruskal–Wallis test (H = 1.98, P = 0.371). Differences in sex distribution were evaluated using the Fisher–Freeman–Halton exact test for the 3 × 2 contingency table (P = 1.000). M, male; F, female; NHP, non-human primate.

***Supplemental Dataset 1*	*Pairwise comparisons of metabolite ratios across retinal phenotypes*.**
Pairwise group comparisons were performed for each metabolite or metabolite ratio across normal, punctate, and drusen-associated eyes using a chi-square test. For each comparison, the “Level” and “–Level” columns indicate the groups being contrasted. The “Score Mean Difference” represents the difference in ranked values between groups, with corresponding standard error (Std Err Dif) and Z statistic. Statistical significance is reported as p-values. Effect sizes are summarized using the Hodges–Lehmann estimator with associated 95% confidence intervals (Lower CL, Upper CL). The “Difference Plot” column reflects the direction and magnitude of the group difference. Positive values indicate higher levels in the first group listed (“Level”), whereas negative values indicate higher levels in the comparison group (“–Level”).
